# Nutritional Status as a Severity Predictor in Critical Pediatric Patients With COVID‐19

**DOI:** 10.1002/ppul.71662

**Published:** 2026-05-14

**Authors:** Gabriela Rupp Hanzen Andrades, Gustavo Rodrigues‐Santos, Jaqueline Rodrigues Robaina, Mariana Bastos Genuíno de Oliveira, Margarida dos Santos Salú, Fernanda de Carvalho Lima, Antonio José Ledo Alves da Cunha, Maria Clara de Magalhães Barbosa, Arnaldo Prata‐Barbosa, Caroline Abud Drumond Costa

**Affiliations:** ^1^ Post Graduate Program in Pediatrics and Child Health of PUCRS ‐ Pontifical Catholic University of Rio Grande do Sul Porto Alegre Rio Grande do Sul Brazil; ^2^ Pontifical Catholic University of Rio Grande do Sul Porto Alegre Rio Grande do Sul Brazil; ^3^ D'Or Institute of Research and Learning (IDOR) Rio de Janeiro Rio de Janeiro Brazil

**Keywords:** children, COVID‐19, mortality, nutritional status, Pediatric Intensive Care Units

## Abstract

**Introduction:**

Nutritional status plays an important part in the health of children and teenagers. It also has an impact on the clinical outcomes of disease situations. This paper's objective is to evaluate the impact of nutritional status in the clinical outcomes of critically ill children diagnosed with COVID‐19.

**Methods:**

This is an observational, longitudinal, and multicentric study, developed between March 2020 and December 2021, in 36 Pediatric Intensive Care Units in Brazil. The patients included were between the ages of 1 month old and 18 years old, with the diagnosis of COVID‐19, confirmed by RT‐PCR, from nasopharyngeal and oropharyngeal swabs, tracheal aspirates, or blood serology for the detection of IgA/IgM/IgG antibodies. Nutritional status was evaluated based on the *z*‐score of the body mass index for age (BMI/A), weight for age (W/A), and stature for age (S/A). The outcomes evaluated included final clinical diagnosis of respiratory syndromes, need for ventilatory support, prolonged length of hospital stay, and mortality. A model of regression linear analysis was used to evaluate the independent association with the nutritional status.

**Results:**

Four hundred and thirty‐two patients were included in the study. The average age was 30 months (IQR, 11−85); 200 (46.3%) were under 24 months old, and 29 (6.7%) had comorbidities. There were 302 (69.9%) patients with average weight, 54 (12.5%) were underweight, 76 (17.6%) were overweight, and 44 (23%) had short stature. The underweight category presented independent association with the outcomes of Acute Respiratory Distress Syndrome (ARDS) (RR 2.12; 95% CI 1.01−4.46; *p* = 0.04), need for invasive mechanical ventilation (IMV) (RR 1.8; 95% CI 1.1–3.1; *p* = 0.02), prolonged length of stay (LOS) (RR 1.5; 95% CI 1.01–2.1, *p* = 0.03), and mortality (RR 8; 95% CI 1.9–36; *p* = 0.005).

**Conclusion:**

**I**ndependent association was identified between low weight in children with COVID‐19 in Pediatric Intensive Care Units and longer length of hospital stay, need for IMV, ARDS and higher risk of mortality.

## 1 Introduction

The infection by the coronavirus SARS‐CoV‐2, which causes COVID‐19, has presented significant variations in its clinical manifestations among different age groups, predominantly mild in pediatric patients [1, 2]. However, the severity of COVID‐19 in children should not be underestimated, especially in the presence of comorbidities, systemic inflammation, or immunological deficiency, factors that may contribute to different outcomes [3].

Malnutrition, including deficit and excess, may have a substantial impact on the outcomes of children with respiratory infections, compromising immune and metabolic responses, and increasing vulnerability to infections [4, 5]. Children in critical states face higher risks of adverse outcomes due to compromised immune and inflammatory responses, higher susceptibility to complications, such as ARDS, and difficulties in supplying adequate nutritional support [6].

Previous studies suggest that adequate nutritional status may be associated with a higher risk of mortality, the need for IMV, and prolonged LOS in Pediatric Intensive Care Units (PICU) [6, 7, 8]. Although the association between inadequate weight and adverse outcomes is well documented in critical pediatric patients, the clinical evidence of this association in pediatric patients with COVID‐19 remains limited.

The relation between obesity and COVID‐19 in adults is already widely documented, with studies indication that inadequate nutritional status is a risk factor for the severity of the disease [9, 10, 11, 12]. Considering that strong evidence in adults already exists, in the pediatric population, this relation is not well elucidated [12].

However, evidence on this association among critically ill pediatric patients with COVID‐19 remains scarce. Therefore, this study aimed to evaluate nutritional status as a predictor of clinical severity in critically ill pediatric patients with COVID‐19, focusing on outcomes such as mortality, need for mechanical ventilation, and prolonged length of stay (LOS).

## 2 Material and Methods

### 2.1 Outlining and Study Location

For this research, patients between 1 month old and 18 years old who were hospitalized with Severe Acute Respiratory Infection (SARI) were included, and a confirmed diagnosis of COVID‐19. In this study, SARI was defined as an acute respiratory infection, with a likely infectious origin, lasting up to 10 days, regardless of severity. Whereas Severe Acute Respiratory Syndrome (SARS) was defined as a case of SARI with severe respiratory discomfort, demanding hospitalization for clinical support. The classification followed the medical records and criteria adopted by the participating PICUs.

Patients without records of weight and stature and those that were not able to be evaluated by the BMI, such as premature or less than 40‐week‐old babies, in adjusted age, were excluded.

This study was approved by the Research Ethics Committee of the Pontifical Catholic University of Rio Grande do Sul (PUCRS), under approval number 4.743.057. The primary study, from which this secondary analysis was derived, was previously approved by the Brazilian National Research Ethics Commission (CONEP), under approval number 3.957.675, dated April 7, 2020.

In the primary study, all participants or their legal guardians provided written informed consent prior to inclusion. This secondary analysis used retrospective and anonymized data, and no additional consent was required, as confirmed by the approving ethics committee.

The research was conducted according to the guidelines of the Strengthening the Reporting of Observational Studies in Epidemiology (STROBE) checklist, to ensure the quality and the transparency of the methods and the presentation of the results.

### 2.2 Data Collection

The collected data included age in months, gestational age for patients up to 24 months old, sex, origin, and preexisting comorbidities. Anthropometric data (weight and height) were obtained retrospectively from patient medical records, corresponding to measurements recorded at PICU admission as part of routine clinical care. The clinical severity was measured by the Pediatric Index of Mortality 3 (PIM3). The clinical outcomes evaluated were final diagnosis (ARDI, SARS, ARDS), mortality, and LOS in the ICU, considering prolonged when over 75% of the sample. Respiratory outcomes, such as the need for oxygen therapy, non‐invasive ventilation (NIV), high‐flow nasal cannula (HFNC), and IMV.

The nutritional status was evaluated by the *z*‐scores of the body mass index for age (BMI/A) and, when available, stature for age (S/A), based on the records of weight and stature. For patients up to 10 years old without a record of stature, BMI/A was used. The analysis was run on Anthro and AnthroPlus software programs of the World Health Organization (WHO), applying the WHO growth cut‐off points. For the statistical analysis, nutritional diagnosis was grouped in three weight categories. Patients classified as thin, severely thin, or low weight for age (W/A) were categorized as “underweight.” The category “normal weight” included patients classified as eutrophic, with risk of overweight and adequate W/A. The category “overweight” encompassed patients classified as overweight, obesity, severe obesity, and elevated W/A. For the analysis of S/A, we grouped patients classified as very short S/A and short S/A in the category “short stature.”

The COVID‐19 diagnosis was confirmed by RT‐PCR from nasopharyngeal and oropharyngeal swabs, tracheal aspirates, or blood serology for the detection of IgA, IgM, and IgG antibodies. Viral and bacterial coinfections were registered through a respiratory panel directed to a comprehensive group of respiratory pathogens, including viruses and bacteria frequently associated with respiratory system infections.

### 2.3 Statistical Analysis

The analysis was conducted using the IBM SPSS Statistics 20 software program. Normality was assessed; categorical variables were described as frequencies and percentages, and compared using chi‐square or Fisher's exact test. Continuous variables were described with median and interquartile range and compared with the Kruskal‐Wallis test. To verify associations between nutritional status and outcomes, a multivariate Poisson regression was performed, adjusted for significant confounding factors in the univariate analysis, adopting *p* < 0.05 as the significance level.

## 3 Results

**Figure 1:**
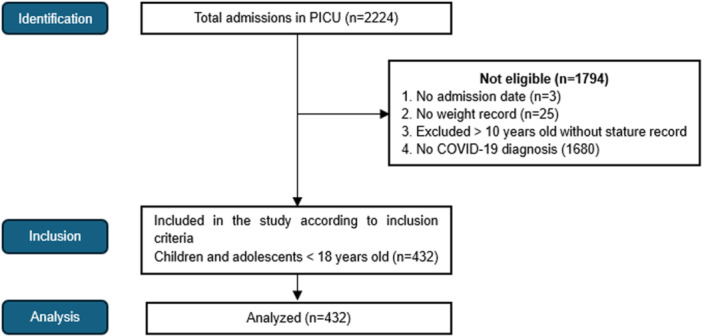
Flowchart of patient selection and inclusion. Patients lacking anthropometric data were excluded. [Color figure can be viewed at wileyonlinelibrary.com]

A total of 432 patients were included in the study. Of these, 68% belonged to the private network, and 63% were from the Southeast region of Brazil. The patient selection flowchart is described in Figure 1.

The median age in months was 30 (IQR, 11–85), of these, 200 (46.3%) were under 24 months old, and 29 (6.7%) had pre‐existing comorbidities, the most common being lung diseases (38, 8.8%), followed by neurological diseases (33, 7.6%) and onco‐hematological diseases (27, 6.7%). The prevalence of patients with pneumonia was the highest, totaling 53.1%. Co‐detection of COVID‐19 with other viruses was identified in 10.2% of the sample. The median of LOS was 5 days (IQR, 3–9), while the mortality rate was 2.3%. Table 1 presents this characterization.

**Table 1:** Clinical and sociodemographic characteristics in patients hospitalized due to COVID‐19 (*N* = 432).

Variables	*N* = 432
Male sex, *n* (%)	224 (51.9)
Age (months), median (IQR)	30 (11–85)
Age group, *n* (%)	
≤24 months	200 (46.3)
25 to 60 months	90 (20.8)
61 to 120 months	75 (17.4)
>120 months	67 (15.5)
Origin, *n* (%)	
Emergency	228 (79.2)
Infirmary	46 (16.0)
Others	14 (4.9)
Type of virus, *n* (%)	
Influenza B	10 (0.5)
H1N1	2 (0.5)
RSV	36 (23.5)
Other viruses	8 (1.8)
Coinfection	44 (10.2)
Comorbidity, *n* (%)	148 (34.3)
Pulmonary disease	38 (8.8)^d^
Neurological or muscular disease	33 (7.6)^d^
Onco‐hematological diseases	27 (6.3)^d^
Other comorbidities	63 (14.6)^d^
≥2 comorbidities, *n* (%)	29 (6.7)
PIM3, median (IQR)	0.8 (0.7–1.7)
LOS, median (IQR)	5 (3–9)
Mortality, *n* (%)	10 (2.3)
Classification S/A^e^, *n* (%)	
Adequate stature	147 (77.0)
Short stature	44 (23.0)
Nutritional status categories	
Underweight	54 (12.5)
Normal weight	302 (69.9)
Overweight	76 (17.6)

Abbreviations: COVID‐19, coronavirus disease 2019; *N*, number; IQR, interquartile range; LOS, Length of Stay; PIM3, Pediatric Index of MorTality 3; RSV, Respiratory Syncytial Virus; S/A, stature‐for‐age, URTI, Upper Respiratory Tract Infections.

^a^ *N* = 288;

^b^ *N* = 303;

^c^ *N* = 427;

^d^ Some patients had more than one comorbidity.

^e^ *N* = 191.

Table 2 presents univariate analysis of the relationship between nutritional status categories and demographic, clinical variables, and outcomes in children with COVID‐19 in critical condition. Underweight patients were predominantly younger than 24 months, while older patients were predominantly overweight (*p* < 0.001). Coinfection with COVID‐19 and Influenza B (*p* = 0.001) or H1N1 (*p* = 0.03) was also more prevalent in the underweight group, as well as the presence of two or more comorbidities (*p* < 0.001).

**Table 2:** Relationship between nutritional status and demographic, clinical variables, and outcomes in patients with COVID‐19.

Variables		*N* = 432		
	Low weight (*n* = 54)	Adequate weight (*n* = 302)	Overweight (*n* = 76)	*p*
Age group				
≤24 months	**40 (74.1)** *	146 (48.3)	14 (18.4)	**< 0.001**
25 to 60 months	8 (14.8)	69 (22.8)	13 (17.1)	
61 to 120 months	4 (7.4)	53 (17.5)	18 (23.7)	
>120 months	2 (3.7)	34 (11.3)	**31 (40.8)** *	
Male sex, *n* (%)	26 (48.1)	162 (53.6)	36 (47.4)	0.520
Virus				
Influenza B	**2 (3.7)** *			**0.001**
H1N1	**1 (1.9)** *			**0.030**
RSV	4 (23.5)	29 (24.8)	3 (15.8)	0.690
Viral coinfection	5 (9.3)	36 (11.9)	3 (3.9)	0.110
Comorbidities, *n* (%)	24 (44.4)	96 (31.8)	28 (36.8)	0.170
≥2 Comorbidities, *n* (%)	**12 (22.2)** *	13 (4.3)	4 (5.3)	**< 0.001**
PIM3	1.7 (0.5‐3.2)	0.8 (0.5‐1.7)	0.8 (0.6‐1.3)	0.620
Respiratory support, *n* (%)				
NIV	7 (13)	28 (9.3)	8 (10.5)	0.690
Days of NIV use, (IQR)	2 (1–4)	2 (1–5)	3 (2–5)	0.560
HFNC	6 (11.1)	27 (8.9)	3 (3.9)	0.270
Days of HFNC use, (IQR)	3 (3–8)	4 (3–5)	4 (4)	0.530
Oxygen therapy	16 (29.6)	126 (41.7)	30 (39.5)	0.240
Days of oxygen therapy use, (IQR)	3 (2–6)	3 (1–5)	4 (2–8)	0.270
IMV	**19 (35.2)** *	47 (15.6)	9 (11.8)	**0.001**
Days of IMV use, (IQR)	16 (4–26)	7 (3–12)	8 (4–13)	0.200
Outcomes				
ARDS	**12 (23.1)** *	26 (8.9)	6 (8)	**0.006**
SARS	2 (3.7)	22 (7.3)	2 (2.6)	0.230
SARI	12 (22.2)	67 (22.2)	17 (22.4)	0.990
MIS‐C	1 (1.9)	25 (8.3)	7 (9.2)	0.220
Prolonged LOS^c^	24 (46.2)	87 (30.1)	22 (30.6)	0.070
LOS, median (IQR)				0.060
Mortality	**6 (11.3)** *	4 (1.3)		**< 0.001**

*Note:* Pearson's chi‐square test or Kruskal−Wallis' test, depending on the distribution of the variables.

Abbreviations**:** ARDS, acute respiratory distress syndrome; COVID‐19, coronavirus disease; HFNC, high‐flow nasal cannula; IQR, interquartile range; IMV, invasive mechanical ventilation; LOS, length of stay; MIS‐C, multisystem inflammatory syndrome in children; *N*, number; NIV, non‐invasive ventilation; NW, Normal weight; OW, overweight; PIM3, Pediatric Index of Mortality 3; SARI, severe acute respiratory infection; RSV, respiratory syncytial virus; S/A, Stature‐for‐age; SARS, severe acute respiratory syndrome; SARS‐COV2, severe acute respiratory syndrome coronavirus 2; UW, underweight.

^a^ *N* = (UW = 44; NW = 215; OW = 44);

^b^ *N* = (UW = 52; NW = 293; OW = 75);

^d^ *N* = (UW = 53; NW = 300; OW = 74);

^c^ *N* = (UW = 52; NW = 289; OW = 72);

* Bold indicates statistically significant results (*p* < 0.05).

Regarding clinical outcomes, underweight patients had a greater need for mechanical ventilation (*p* = 0.001) and a higher prevalence of ARDS (*p* = 0.006). Mortality was significantly associated with underweight patients (*p* < 0.001), while no deaths were recorded in the overweight group.

In the analysis of linear regression, underweight patients presented higher necessity for IMV (RR 1.8; 95% CI 1.1–3.1; *p* = 0.020). Low weight patients presented independent association with ARDS (RR 2.12; 95% CI 1.01–4.46; *p* = 0.040), prolonged LOS (RR 1.5; 95% CI 1.01–2.1; *p* = 0.030), and mortality (RR 8; 95% CI 1.9–36; *p* = 0.005) (Table 3).

**Table 3:** Multivariate analysis of the relationship between nutritional status and clinical outcomes in patients admitted to PICU with COVID‐19.

		Nutritional study category					S/Aclassification		
Variables	Low weight			Overweight			Short stature for age		
	RR	95% CI	*p*	RR	95%CI	*p*	RR	95% CI	*p*
IMV	1.83	1.09−3.08	**0.020** *	0.56	0.27‐1.15	0.12	1.29	0.77−2.18	0.32
Prolonged LOS	1.47	1.02 −2.11	**0.030** *	0.88	0.58‐1.34	0.56			
ARDS	2.12	1.01‐4.46	**0.040** *	0.76	0.32‐1.77	0.52			
Mortality	8.20	1.86−36.02	**0.010** *						

*Note:* Adjusted for age group, comorbidities, and viral coinfection. Reference categories = Adequate stature and normal weight. It was not possible to estimate mortality in the overweight category because of the insufficient number of cases. Poisson regression.

Abbreviations: ARDS, acute respiratory distress syndrome; CI, confidence interval; IMV, invasive mechanical ventilation; LOS, length of stay; RR, relative risk; S/A, stature‐for‐age.

* Indicates statistically significant results (*p* < 0.05).

## 4 Discussion

In this multicenter cohort study, low weight in pediatric patients with COVID‐19 was independently associated with an increased risk of developing ARDS, need for IMV, prolonged length of hospital stay, and mortality, reinforcing the importance of nutritional status as a predictor of clinical severity in critically ill pediatric patients.

The association between low weight and worse outcomes in the PICU may be related to an elevated catabolic state, low nutritional intake, and decreased nutrient absorption, which lead to loss of muscle mass, impaired respiratory function, and increased susceptibility to infections [13, 14, 15]. It is believed that children with low weight may have a compromised immune system, thus increasing the risk of developing respiratory infections, which in turn may trigger ARDS [16]. The present study showed that children with low weight have approximately twice the risk of developing ARDS. A previous study reported the presence of ARDS in approximately 60% of children requiring intensive care, highlighting the severity of the condition in these patients [17].

In addition, underweight patients had an 83% higher risk of requiring IMV, possibly due to reduced lung capacity related to muscle depletion [18]. According to a recent meta‐analysis, malnutrition is associated with a higher need for IMV in critically ill pediatric patients, including those with severe bronchiolitis. This association may be explained by lower lung capacity in underweight children, predisposing them to a compromised respiratory response [7]. Short stature may reflect chronic malnutrition or underlying diseases that impair growth and increase vulnerability to adverse outcomes. However, in the present study, no independent association was found between short stature and clinical outcomes after adjustment for confounding factors.

Short stature was also identified as an additional risk factor for respiratory outcomes, reflecting chronic malnutrition or underlying diseases that affect growth and increase vulnerability to respiratory complications. Although no independent association was observed between short stature and the need for mechanical ventilation, it is important to consider that this condition may indicate chronic malnutrition or prolonged diseases, reflecting an insufficient intake of essential nutrients over time, which may negatively impact growth and development [19].

In contrast to malnutrition, obesity has also been described in studies as a worsening factor for ARDS, due to the chronic inflammatory state that can increase cytokine production and contribute to the development of the condition 20, 21, 22. Overweight children may present immunological imbalances, such as alterations in cytokine and protein concentrations and in the function of immune cells, which increases susceptibility to infection and pathogenicity of SARS‐COV2 [20, 23]. Although our study did not find a significant association between obesity and adverse outcomes, previous studies in adults suggest that excess weight increases the need for IMV, possibly due to additional adipose tissue, which interferes with gas exchange and increases resistance during breathing [21, 22, 23, 24].

Low weight has been consistently associated with prolonged LOS in patients admitted to the PICU. Children in this condition are at greater risk of clinical deterioration due to increased metabolic demands and challenges in ensuring adequate nutritional support, which may negatively impact their prognosis. One of the first studies evaluating the relationship between nutritional status and clinical outcomes in hospitalized patients with COVID‐19, conducted in a cross‐sectional design, showed that malnutrition was associated with increased LOS and mortality risk. However, this study included both children and adults, limiting the ability to assess the impact exclusively in the pediatric population [10]. Other studies assessing nutritional risk at hospital admission in patients with COVID‐19 have also reported an association between higher nutritional risk and prolonged LOS, although without classifying outcomes according to anthropometric nutritional status, which limits direct comparisons with our findings [11, 25].

Regarding mortality, underweight patients demonstrated an 8.2‐fold increased risk compared to other weight categories. The risk associated with being underweight is a recognized finding in the literature [7, 26]. Corroborating this result, a systematic review concluded that malnutrition is associated with an increased incidence of mortality in this population [27, 28]. In patients with COVID‐19, previous studies have reported associations between both overweight and malnutrition and mortality in adults. However, among children, there is still a lack of robust studies that can fill this gap [10, 27, 28].

This study has limitations, including the potential for measurement bias due to its multicenter nature, as each hospital used its own recording systems without complete standardization of variables. In addition, the clinical condition of the patients may have impacted the collection of anthropometric data at admission. The use of indicators such as BMI/A and W/A represents another limitation, as these do not provide detailed information on body composition, which could allow a more accurate assessment of nutritional status. Furthermore, mortality data should be interpreted with caution due to the wide confidence intervals observed and the relatively low mortality incidence in the sample (2.3%), which may limit the statistical power to accurately estimate this outcome. This limitation may influence the precision of the results regarding the impact of low weight on mortality. Additionally, regional socioeconomic disparities in Brazil may represent a limitation, as they can influence the distribution of nutritional status across different populations. Among the strengths, the multicenter design stands out, including PICUs from different regions of Brazil, which increases the representativeness of the sample and strengthens the robustness of the findings.

## 5 Conclusion

In conclusion, nutritional status was a significant predictor of clinical severity in critically ill pediatric patients with COVID‐19. Low weight, in particular, was independently associated with adverse outcomes, including increased risk of ARDS, prolonged hospitalization, need for mechanical ventilation, and mortality. These findings highlight the importance of incorporating nutritional assessment, prevention, and management as part of standard care for pediatric patients with COVID‐19, aiming to improve prognosis and reduce associated complications.

## Author Contributions

**Gabriela Rupp Hanzen Andrades:** conceptualization, investigation, methodology, validation, visualization, writing – review and editing, data curation, writing – original draft, project administration. **Gustavo Rodrigues‐Santos:** investigation, validation, visualization, project administration, data curation, conceptualization, software. **Jaqueline Rodrigues Robaina:** investigation, formal analysis, data curation, validation, visualization. **Mariana Bastos Genuíno de Oliveira:** investigation, visualization, data curation. **Margarida dos Santos Salú:** investigation, validation, visualization. **Fernanda de Carvalho Lima:** investigation, validation, visualization. **Antonio José Ledo Alves da Cunha:** investigation, validation, visualization. **Maria Clara de Magalhães Barbosa:** investigation, validation, visualization. **Arnaldo Prata‐Barbosa:** conceptualization, investigation, writing – review and editing, validation, visualization, methodology, software, project administration, data curation, supervision. **Caroline Abud Drumond Costa:** conceptualization, investigation, writing – original draft, writing – review and editing, validation, visualization, methodology, project administration, data curation, supervision.

## Collaborating Authors, from the Brazilian Research Network in Pediatric Intensive Care

Ana Carolina Miranda Carvalho F F Souza–Oeste D'Or Hospital. Ana Paula Novaes Bellinat–Martagão Gesteira Hospital. Bárbara Carvalho Santos dos Reis–Niterói Hospital Complex; Unimed Leste Fluminense. Carolina Friedrich Amoretti–Prof. Edgard Santos University Hospital. Cibelle Teixeira da Siva Borges–São Camilo Cura D'Ars Hospital. Daniel Hilário Santos Genu–Getúlio Vargas State Hospital. Elaine Augusta das Neves Figueiredo–Unimed Pediatric Hospital. Fábio Zattar Guerios–Aliança Hospital. Fabíola Peixoto Ferreira La Torre–Sino‐Brazilian Hospital. Felipe Rezende Caino de Oliveira–Alvorada Moema Hospital. Flavia Andrea Krepel Foronda–Syrian Lebanese Hospital. Igor Bromonschenkel Brandão–Niterói D'Or Hospital. João Henrique Garcia Cobas Macedo–Copa D'Or Hospital. José Colleti Jr.–Assunção Hospital. Lucas Pulcheri–Rios D'Or Hospital. Lucio Flavio Peixoto de Lima–Sepaco Hospital. Maria Carvalho Laborne Valle–Jutta Batista Hospital. Melissa de Lorena Jacques–Quinta D'Or Hospital. Natália Almeida A Silva Rodriguez Castro–Children and Maternity Hospital (FMSJRP). Natalia Martins Lima de Lucena–Mount Klinikum Hospital. Orlei Ribeiro de Araújo–GRAAC. Patrícia Silva de Vasconcellos Lara–Da Luz Hospital. Paula Marins Riveiro–Caxias D'Or Hospital. Raquel de Seixas Zeitel–Pedro Ernesto University Hospital. Roberto Sapolnik–St. Rafael Hospital. Rosana Novais de Carvalho–St. Izabel Hospital (Santa Casa). Sérgio D'Abreu Gama–Nova Iguaçu Pediatric Emergencies. Simone Camera Gregory–Children's State Hospital. Thiago Peres da Silva–Real D'Or Hospital. Vivian Botelho Lorenzo–Couto Maia Hospital. Zina Maria Almeida de Azevedo–Fernandes Figueira Institute.

## Conflicts of Interest

The authors declare no conflicts of interest.

## Data Availability

The data that support the findings of this study are available from the corresponding author upon reasonable request.
